# Comparison of Additively Manufactured Polymer-Ceramic Parts Obtained via Different Technologies

**DOI:** 10.3390/ma17010240

**Published:** 2024-01-01

**Authors:** Katarzyna Jasik, Janusz Kluczyński, Danuta Miedzińska, Arkadiusz Popławski, Jakub Łuszczek, Justyna Zygmuntowicz, Paulina Piotrkiewicz, Krzysztof Perkowski, Marcin Wachowski, Krzysztof Grzelak

**Affiliations:** 1 Institute of Robots & Machine Design, Faculty of Mechanical Engineering, Military University of Technology, Gen. S. Kaliskiego 2 St., 00-908 Warsaw, Poland; katarzyna.jasik@student.wat.edu.pl (K.J.); jakub.luszczek@wat.edu.pl (J.Ł.); marcin.wachowski@wat.edu.pl (M.W.); krzysztof.grzelak@wat.edu.pl (K.G.); 2 Institute of Mechanics and Computational Engineering, Faculty of Mechanical Engineering, Military University of Technology, Kaliskiego 2 St., 00-908 Warsaw, Poland; danuta.miedzinska@wat.edu.pl (D.M.); arkadiusz.poplawski@wat.edu.pl (A.P.); 3 Faculty of Materials Science and Engineering, Warsaw University of Technology, 141 Woloska St., 02-507 Warsaw, Poland; justyna.zygmuntowicz@pw.edu.pl (J.Z.); paulina.piotrkiewicz.dokt@pw.edu.pl (P.P.); 4 Łukasiewicz Research Network, Institute of Ceramics and Building Materials, 8 Cementowa Street, 31-983 Krakow, Poland; k.perkowski@icimb.pl

**Keywords:** additive manufacturing, microstructural analysis, mechanical properties of ceramics

## Abstract

This paper aims to compare two ceramic materials available for additive manufacturing (AM) processes—vat photopolymerization (VPP) and material extrusion (MEX)—that result in fully ceramic parts after proper heat treatment. The analysis points out the most significant differences between the structural and mechanical properties and the potential application of each AM technology. The research revealed different behaviors for the specimens obtained via the two mentioned technologies. In the case of MEX, the specimens exhibited similar microstructures before and after heat treatment. The sintering process did not affect the shape of the grains, only their size. For the VPP specimens, directly after the manufacturing process, irregular grain shapes were registered, but after the sintering process, the grains fused, forming a solid structure that made it impossible to outline individual grains and measure their size. The highest compression strength was 168 MPa for the MEX specimens and 81 MPa for the VPP specimens. While the VPP specimens had half the compression strength, the results for the VPP specimens were significantly more repeatable.

## 1. Introduction

Since the second half of the 20th century, significant advancements have been made in ceramic-based materials, especially since the introduction of high-purity synthetic raw materials for their production [1]. A significant development in the case of the production of ceramic parts was the usage of AM technologies, which allow for additive, layer-by-layer manufacturing and simplifying the production of complex structures [2,3,4,5]. While polymers and metals dominate the field of AM, there is increasing utilization of less common materials, such as various types of ceramics [6]. Using AM technologies in ceramic components production offers new possibilities and can significantly alleviate the issues encountered with conventional manufacturing methods. Additionally, an AM approach allows for the production of complex geometries [7], which is very difficult in conventional production processes, especially in the case of ceramics. In most cases, ceramic AM materials are available in the form of composites consisting of ceramic particles and an organic, polymeric matrix [8]. To obtain fully ceramic parts, the printed components need to be subjected to additional processing, including extraction, in which the polymer is removed through rinsing, and finally by high-temperature sintering [9,10,11]. 

The first technology that allowed for the AM of ceramic parts was VPP via the stereolithography (SLA) process [12]. Initially, highly concentrated ceramic suspensions were employed, containing up to 65% volume fraction of silica, aluminum oxide, and silicon nitride. The success of the process using such materials depended on the appropriate rheological properties of the ceramic suspension, particularly the viscosity and long-term stability. In the further development of AM technologies, there were also a group of materials available that allowed for obtaining ceramic materials. Based on the available literature analysis, a table of contribution (Table 1) was prepared and is shown below. 

Despite significant progress in the selection of functional ceramic materials, the optimization of processing parameters, and post-processing, there are still barriers to the wider application of 3D printing in ceramic production. Such an issue is mostly caused by a lack of data about the performance properties of ceramic parts obtained by means of different AM technologies. Mass industrial production can be challenging, and larger-sized ceramic components (e.g., several meters) remain difficult to produce using 3D printing due to the characteristic high brittleness and low coefficient of material expansion. That is why it is very crucial to deeply analyze and compare the basic, microstructural phenomena that occur after every step of ceramic material processing, including the AM process, debinding, and sintering. In this research, two AM technologies were taken into account—VPP and MEX—to allow for additional comparisons of the obtained ceramic materials. Such an analysis would fulfill the main aim of this work, which is to compare the properties of the obtained ceramic parts and indicate the most specific pros and cons to point out a potential application of each of the parts obtained via the VPP and MEX technologies.

## 2. Materials and Methods

### 2.1. Materials for the Research

Two types of composite materials with a polymer matrix and ceramic reinforcement were utilized for the research. The first one consisted of aluminum oxide (Al_2_O_3_) in the form of a ceramic polymer filament (Spectrum Filaments, Pęcice, Poland) with a diameter of 1.75 mm. The main properties of this material are presented in Table 2 and Table 3.

The second material used was a photosensitive composite resin, Ceramics Resin Form X (Formlabs, Somerville, MA, USA). This material is a photopolymer filled with silica, which, after being properly heated at a specific temperature, allows for the production of fully ceramic parts. Selected parameters provided by the manufacturer for the printed parts before the sintering process are presented in Table 4, while the properties of the parts after processing are listed in Table 5.

### 2.2. Manufacturing Process

Based on the EN 843-4:2005 [46] standard, four types of cylindrical specimens were designed in the SolidWorks (Dassault Systemes, Vélizy-Villacoublay, France, v. 2019) software. The specimens for the structural analysis had a diameter of 20 mm and a height of 15 mm, while the parts for the compression testing had a diameter of 10 mm and heights of 5, 10, and 15 mm. The finalized 3D models of the designed components were saved in the STL file format and imported into the Computer-Aided Manufacturing (CAM) software dedicated to preparing the AM processes—Simplify 3D (Simplify 3D LCC., Cincinnati, OH, USA, Version 4.0.1). The complete manufacturing process encompassed AM (green parts), extraction—only for printed parts using FDM/FFF (brown parts), as well as sintering (ceramic parts). To produce specimens using the FDM/FFF method, a Creality CR-10S (Creality, Shenzhen, China) 3D printer was used. Manufacturing using this method was hindered by the significant brittleness of the filament. To minimize the impact of the filament feeding system on the material, modifications were made to this system (referred to as the extruder mechanism). The compression spring of the filament guide bearing was removed, allowing for manual adjustment of the compression level to enable filament insertion into the printing head without causing substantial damage. The AM parameters for FDM/FFF were adjusted for the selected material and device model as follows:

- Nozzle temperature: 175 °C,
- Layer height: 0.2 mm,
- Nozzle diameter: 0.4 mm,
- Print speed: 5 mm/s,
- Infill density: 100%,
- Number of outer shells: 2.

Each specimen was printed individually to ensure even distribution of the softened material by the device, aiming to minimize the occurrence of any structural defects. After the FDM/FFF process, the specimens were subjected to additional extraction of bonding solvent from Al_2_O_3_ by fully immersing the specimens in acetone. The container with the parts was covered to prevent the evaporation of the acetone and placed in a laboratory dryer. The specimens were then heated according to the manufacturer’s recommendations at a temperature of 42 °C for 24 h. All specimens were weighed before and after extraction to determine the mass loss. For the Al_2_O_3_ material, the percentage of mass loss after 24 h of extraction should not exceed 9.8%. The obtained results fell within the range (with the highest loss being 6.72%); thus, all the components underwent further processing.

The Form 2 printer by Formlabs (Formlabs, Somerville, MA, USA) was used for SLA printing. The printing parameters were specified by the manufacturer and were as follows:

- Layer thickness: 0.1 mm,
- Resin type: Photopolymer methacrylate resin,
- Laser spot size: 140 μm,
- Exposure time: 15 s,
- Print speed: 50 mm/h.

The specimens were produced directly on the build platform without the need for support. Subsequently, they were rinsed with isopropyl alcohol to prevent any damage to the material’s structure. Unlike other materials used in SLA printing, ceramics do not require additional UV exposure. Therefore, the rinsed specimens were subjected to drying before proceeding with further processing stages.

### 2.3. Sintering Process

The sintering of the printed specimens was carried out according to the manufacturers’ recommendations. For SLA, a Magma Therm MT-1300-5-B2 (Danlab, Białystok, Poland) furnace was used at the maximum sintering temperature of 1271 °C. All processing parameters are shown in Table 6.

For FDM/FFF, the sintering was performed at a temperature of 1540 °C in a Nabertherm HT 08/18 furnace (Nabertherm, Lilienthal, Germany) equipped with Kanthal Super heating parts. The sintering program is presented in Table 7.

The sintering process enabled the removal of polymer material components from the volume. Upon completion of all manufacturing stages, fully ceramic components were obtained, which were subsequently prepared for detailed structural examinations.

### 2.4. Testing Methods

In order to prepare all the specimens for further analysis, they were cut along the printed layers (along the Z-axis). Subsequently, half of the cut specimens were mounted in resin and ground using abrasive water paper with the following grit sizes: 240, 320, 500, 800, and 1200. The physical properties of the specimens were measured using the Archimedes method. The density was determined using an automatic helium pycnometer (AccuPyc 1340 II by Micromeritics, Norcross, GA, USA). Measurements were conducted in a cylindrical measurement chamber with a diameter of 19 mm and a height of 39.8 mm in two stages: 10 purges and 700 measurement cycles at a filling pressure of 0.13 MPa for both stages. The volume of the powders was measured using gas displacement, applying the volumetric–pressure relationship of Boyle’s law. During the measurement, the powder was tightly enclosed in a container of known volume. It was then placed in the specimen chamber. Helium gas was introduced into the chamber during the measurement and subsequently expanded into a second empty chamber of known volume. The observed pressure after filling the measurement chamber and the pressure vented to the expansion chamber were recorded during the experiment. The volume was determined based on the mentioned measurements. Density was defined as the ratio of the specimen’s mass to the determined volume. 

For phase composition analysis, a Rigaku Mini Flex II diffractometer (Rigaku, Tokyo, Japan) with CuKα radiation and a wavelength of λ = 1.5406 Å was used. The following parameters were applied:

- 2θ angle range: 20–100° (for Series 1, 3, and 5); 15–60° (for Series 2 and 4)
- Voltage: 30 kV,
- Current: 15 mA,
- Step size: 0.03°,
- Counting time: 2 s.

The analysis was performed on flat parallel surfaces. The MDI JADE 8.5 software (Materials Data, Inc., Livermore, CA, USA) was used for phase composition analysis. The interpretation of the obtained results was based on the standard X-ray database ICDD PDF + 4 2022. The morphology and chemical composition analysis of the produced specimens were examined using a Jeol JSM–6610 (Jeol, Tokyo, Japan) scanning electron microscope (SEM) equipped with the energy dispersive spectroscopy (EDS) module. Subsequently, the particle size of the powder was calculated from the SEM images. 

Stereological analysis of SEM images was carried out to determine the grain size depending on the use of technologies. For this purpose, the MicroMeter v.086b [47,48] software was used. The analysis supplied information on the sizes of the Al_2_O_3_ and SiO_2_ grains. Stereological analysis was carried out to determine the grain size depending on the use of technologies. For this purpose, the MicroMeter v.086b (Warsaw University of technology, Warsaw, Poland, version 086b) [47,48] software was used. The analysis supplied information on the size of the Al_2_O_3_ and SiO_2_ grains.

Hardness measurements were performed using the Vickers method [45] on a DuraScan 70 hardness tester (Struers, Copenhagen, Denmark). The measurements were conducted using a diamond pyramid-shaped indenter with a vertex angle of 120°. Five measurements were taken for each specimen, and two extreme values were discarded prior to calculations. The distance between the individual measurements was three times greater than the diameter of the indentation to prevent the mutual influence of the results. The last of the tested structural properties was roughness, which was determined using a digital optical microscope Keyence VHX-7000 (Keyence, Osaka, Japan). For each analyzed specimen, two points were selected along a straight line passing through the layers of the individual specimens. 

**Figure 1:**
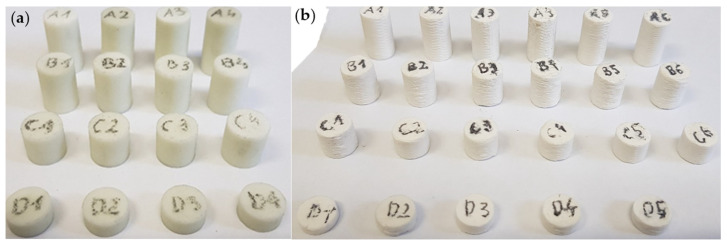
AM specimens for compression testing obtained with (**a**) SLA technology and (**b**) FDM/FFF technology.

In the final step, an investigation of the ceramic material obtained by both AM methods was made. The analysis involved conducting static compression tests to determine stress–strain curves and calculate basic strength parameters. The compression tests were conducted using the Zwick/Roell KAPPA DS 50 (Zwick, Ulm, Germany) universal testing machine equipped with an electromechanical drive and a 50 kN force measurement head. Strains were measured using a Phantom V12 Vision Research video extensometer (Vision Research, Inc., NJ, USA). Compression tests were performed at a traverse displacement rate of 2 mm/min. The specimens of the test materials were produced using the SLA and FDM methods. The specimens were printed as cylinders with a diameter of approximately 10 mm and heights of 5 mm (D1–D5 in Figure 1), 10 mm (C1–C6 in Figure 1), 15 mm (B1–B6 in Figure 1), or 20 mm (A1–A6 in Figure 1). Figure 1 presents exemplary sets of specimens. After printing, the specimens underwent a heat treatment process at two different temperatures for each sample’s series: 1271 °C and 1540 °C. A minimum of 5 specimens were tested for each type.

## 3. Results and Discussion

All investigations were conducted on seven different specimen series. The properties of the printed parts, as well as the influence of sintering temperature, were compared using two different methods. Table 8 presents the designations and types of the tested specimens. The specimen descriptions in all the studies were consistent with the aforementioned table.

### 3.1. Density Analysis

In the first stage of the research, the density of all produced specimens was determined using the pycnometer method, taking into account the non-zero volume of the measuring vessel. The results of the investigation are presented in Table 9.

Specimens produced using the FDM/FFF method exhibited a 35% higher density compared to their SLA counterparts (2.576 g/cm^3^ for FDM/FFF and 1.721 g/cm^3^ for SLA). The densities of both specimens increased after sintering at lower temperatures, reaching 2.306 g/cm^3^ for SLA and 4.057 g/cm^3^ for FDM/FFF. A visible densification, especially in the alumina samples, is related to the grain growth, which indicated a typical densification mechanism in ceramic materials related to the diffusion of the material in the whole volume of the sample part. The visible densification mechanism was described by Bordia et al. [49] and is related to the material transport from the grain boundary or particles’ surface to the neck surface (sink). However, the density values slightly decreased after sintering at a higher temperature, measuring 2.250 g/cm^3^ for SLA and 3.920 g/cm^3^ for FDM/FFF. This could be due to the material redistribution of the surface particles (bonding mechanism) [49] and is related to the material transport from the grain boundary or particles’ surfaces to the neck surface (sink). The density of the aluminum oxide filament material provided by the manufacturer was 2.576 g/cm^3^ before sintering and 3.85–3.96 g/cm^3^ after sintering. The pre-sintering density was slightly different from the pycnometer measurement. On the other hand, the resin manufacturer for SLA provided a post-thermal treatment density value of 1.9 g/cm^3^, which was lower than the density calculated using the pycnometer method. The discrepancies in density could be attributed to insufficient powder desorption before measurement. Additionally, the results may have been affected by surface contamination of the test powder during preparation for measurement. The average pore volume was highest for Specimen Series 2, with a value of 0.4081 ± 0.0013 cm^3^/g, and lowest for Specimen Series 5, which was 0.7351 ± 0.0004 cm^3^/g. Both FDM/FFF and SLA specimens exhibited increased pore volume at the lower sintering temperatures, while increasing the temperature decreased the average pore value. 

### 3.2. Microstructural Investigation

**Figure 2:**
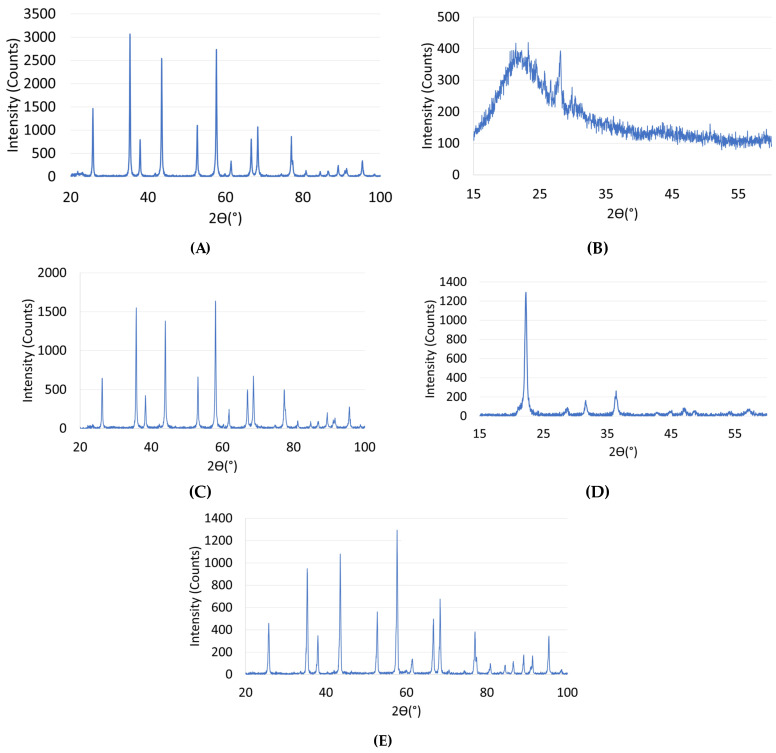
X-ray diffractogram (XRD) for all tested AM specimens. (**A**–**E**) descriptions are shown in Table 8.

Upon analyzing the obtained diffraction patterns for all tested specimens (Figure 2) in their raw state, as well as after processing, it was observed that the materials were single-phase, and the extraction and sintering processes did not result in the emergence of new phases. This indicates the high purity of the starting powders. For the materials fabricated via the FDM/FFF method (Figure 2A,C,E), no effect of the processing and sintering on the formation of new phases in the material was observed. Phase composition analyses of Series 1, 3, and 5 specimens revealed the presence of reflections originating from hexagonal Al_2_O_3_. What is more, in the case of FDM/FFF, the XRD profile revealed a characteristic peaks crystalline alpha (α)-alumina [50] (25.5°,35°,43°, 52.5°, 57.5°, and 68°) that proves the high purity and stability at all stages of temperature used during the processing of the green and brown parts.

**Figure 3:**
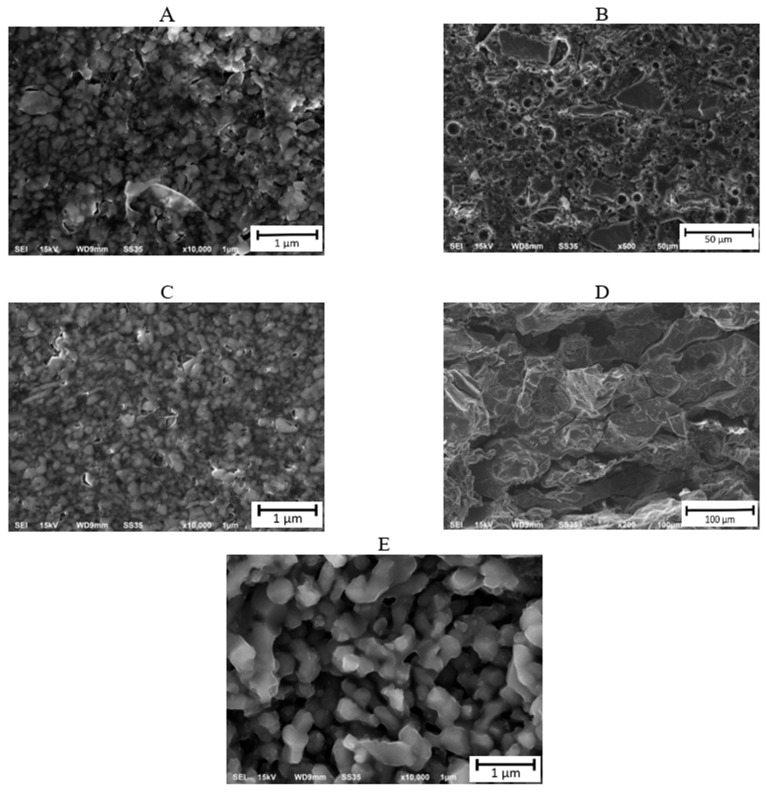
SEM fractures of all tested AM specimens. (**A**–**E**) descriptions are shown in Table 8.

When comparing the results for the SLA method, it was observed that the diffraction pattern for the printed specimens (Figure 2B) differed from the pattern of the sintered specimen (Figure 2D). The raw sample diffractogram indicates the predominance of the amorphous nature of the fabricated material. The visible peak in the spectrum, however, can be attributed to the monoclinic SiO_2_ phase. Whereas the phase analysis of the sample after the sintering process revealed the presence of tetragonal SiO_2_ in the material, the spectrum itself indicated the polycrystalline nature of the sample. This indicates changes in the material structure and SiO_2_ phase transformation that occurred during sintering. The obtained results are in agreement with available literature data, which indicate the possibility of SiO_2_ phase transformations under the influence of high temperatures or pressures [51]. In the next step, microscopic observations were conducted on the aluminum oxide and silicon oxide specimens (Figure 3). Analysis of the obtained images revealed that the aluminum oxide powder tended to form agglomerates and exhibited a fairly regular morphology with rounded edges and mostly spherical shapes. Extraction and sintering did not alter the shape of the particles, only their size. The particle size increased with higher temperatures. In comparison, silicon oxide had grains with slightly different shapes. Before sintering, the grains exhibited irregular morphology. SEM images showed that the powder grains had various shapes, including oval and rectangular, with irregular forms. Numerous voids were also visible. However, after sintering, the structure became solid, grain boundaries were not visible, and the number of voids decreased.

**Figure 4:**
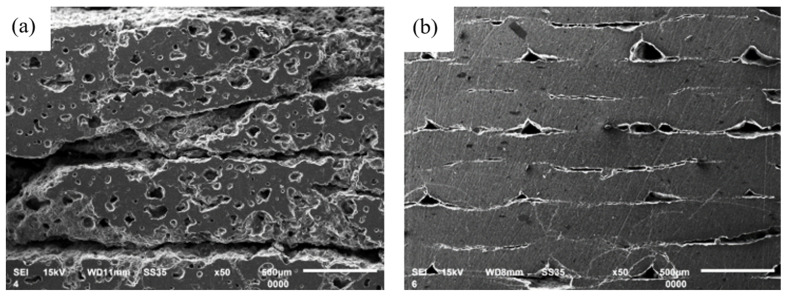
SEM images with visible porosity distribution: (**a**) Specimen Series 4—sintered SLA (**b**) Specimen Series 5—sintered FDM/FFF.

On the SEM images (Figure 4) at a higher magnification, the distribution of porosity within the examined parts can be observed. After the sintering process, Specimen Series 4, printed using the SLA method, exhibited significantly higher porosity compared to Specimen Series 5, produced using the FDM/FFF technique. What is more, in the specimens obtained via the SLA method, there are numerous visible defects between the layers, which significantly affect the possibility of crack initiations occurring. Such a phenomenon could be very dangerous, especially because Wu et al. [52] proved that the cracking mechanism mainly initiates from the edge of the surface and propagates toward the center region of the Si-based parts. 

**Figure 5:**
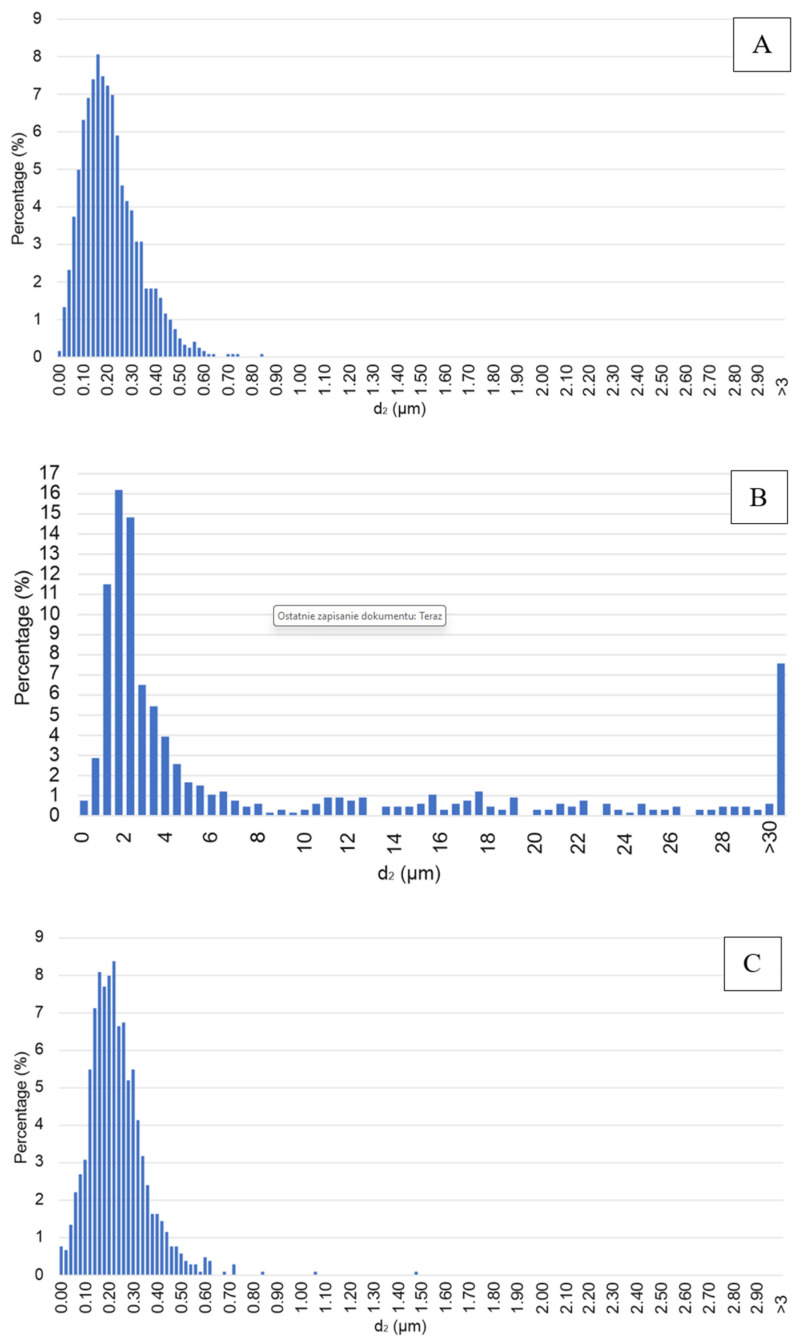

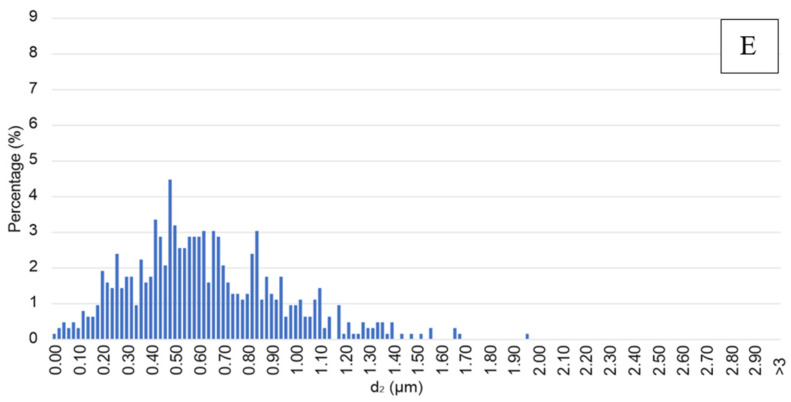
Histograms of the average grain size distribution of all tested AM specimens. Descriptions (**A**–**C**,**E**) are made accordingly to Table 8.

Specimen Series 4 had a higher number of voids, which were unevenly distributed throughout the entire structure of the part. On the other hand, for Specimen Series 5, the voids occurred between two distributed layers, and their number was significantly lower. For deeper analysis, stereological tests were conducted. The analysis involved the manual detection of particles from scanning electron microscope photographs. Then, following a series of image transformations, a binary image of the particles was acquired and analyzed using the Micrometer software (Warsaw University of technology, Warsaw, Poland, version 086b). On the basis of the obtained results, the average equivalent diameter d_2_ was determined for each of the analyzed series, along with its percentage distribution diagrams. The average equivalent diameter d_2_ is a parameter that describes the diameter of a circle with the identical surface area as the corresponding non-spherical surface area of the grain. Specimens of Series D produced by the SLA method after sintering were excluded from the analysis due to the inability to detect proper grain boundaries. Average equivalent diameter distributions for aluminum oxide and silicon oxide after printing are presented in histograms (Figure 5). Analysis of the histograms for the Al_2_O_3_ samples before sintering (Figure 5A,C) showed a symmetrical character with a distinct maximum. For both series, the determined values of the average equivalent diameter d_2_ were in the range of 0.01 to 0.70 μm. However, in the case of Al_2_O_3_ samples after the sintering process (Figure 5E), the increase in the percentage of greater average equivalent diameter d_2_ values can be seen in the graph. The values of d_2_ were in the range of 0.01 to 1.70 μm for this series. The obtained results indicate the occurrence of Al_2_O_3_ grain growth during sintering. For the SiO_2_ sample after printing, a differentiation in terms of particle size in the specimen structure is evident [53]. The average equivalent diameter values obtained in this case were higher than for the samples produced from Al_2_O_3_, with a significant percentage of particles with the d_2_ values in the range of 1 to 4 μm as well as above 30 μm.

The conducted analysis corroborates the determined values of the average equivalent diameter for each of the analyzed series shown in Table 10. 

**Figure 6:**
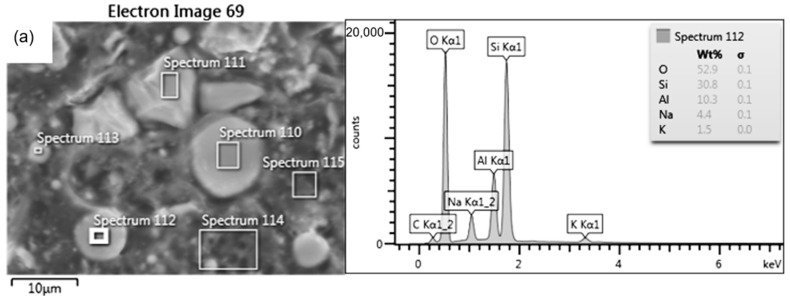

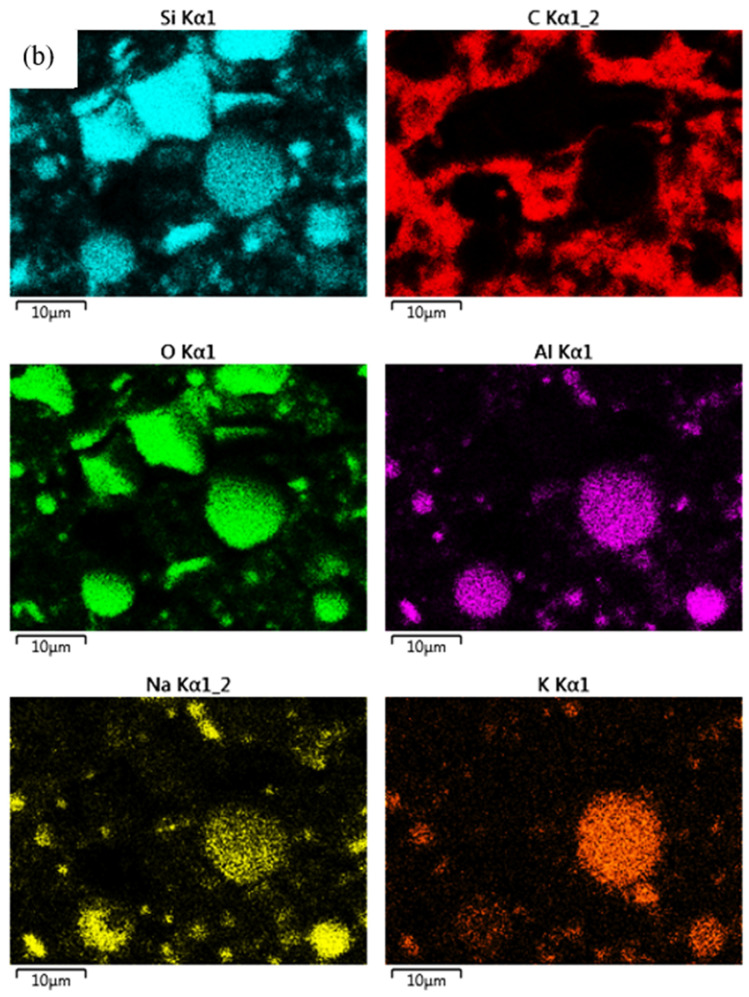
Chemical composition results for Specimen Series 2: (**a**) EDS, (**b**) element distribution map.

**Figure 7:**
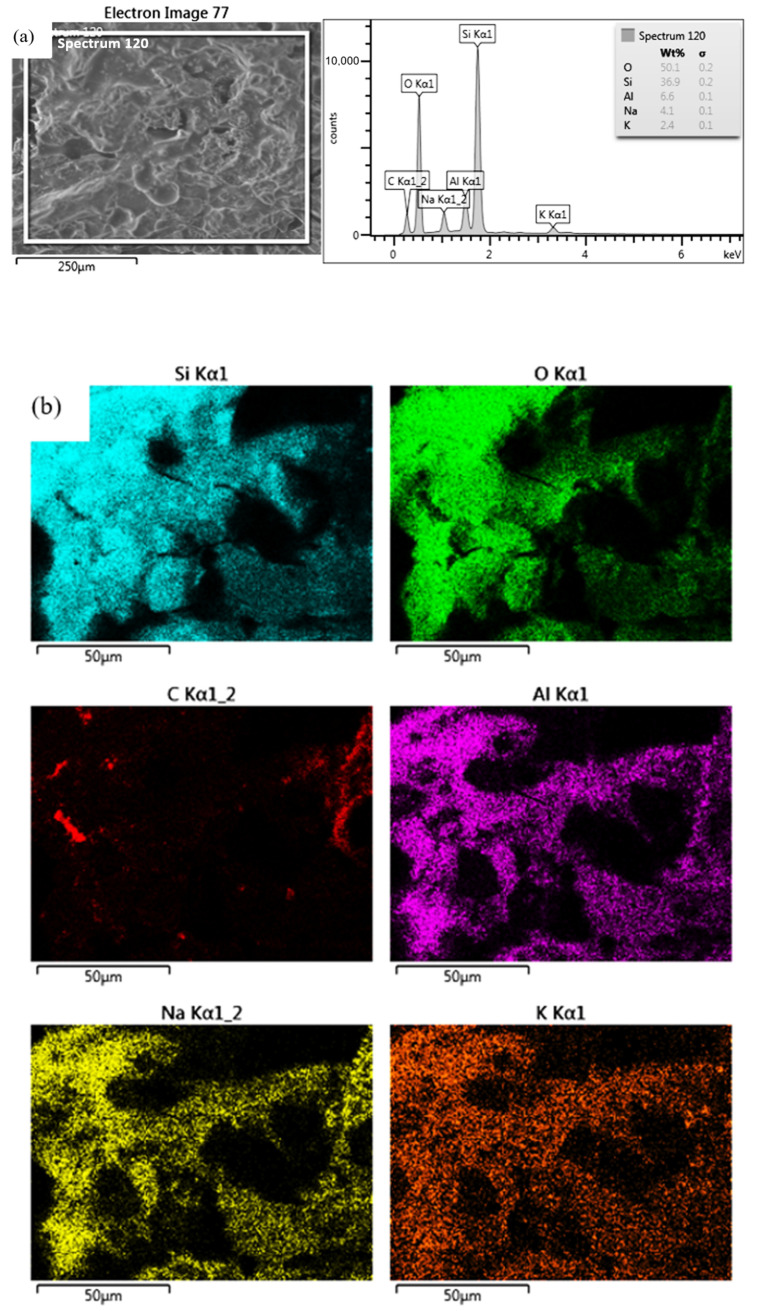
Chemical composition results for Specimen Series 4: (**a**) EDS, (**b**) element distribution map.

For the FDM/FFF specimens (Specimen Series 5 in Table 10), sintered at a temperature of 1540 °C, the grain growth was more than twofold, reaching 0.5 μm. On the other hand, the grain size of silicon oxide produced by SLA, which measured 2.0 μm, was significantly larger than the grain size of aluminum oxide obtained by the FDM/FFF method, both before and after post-processing. The results of the EDS analysis and the chemical composition of each specimen are shown in Table 11. Samples made of aluminum oxide confirmed the high purity of the powders. However, for silicon oxide, the observation revealed the presence of additional elements such as aluminum, sodium, potassium, and calcium. A presence of different elements in the material structure could be related to the lack of a melt phase under normal circumstances in the Si-based ceramic materials [35]. Additional images registered during the EDS analysis of SLA specimens are shown in Figure 6 and Figure 7.

The manufacturer of the composite intended for the SLA process does not specify the exact composition of the powder but states that it is a photocurable resin filled with silica. Therefore, additional elements could be present in the material. As for the presence of calcium in both materials after sintering at a temperature of 1540 °C, this was most likely due to contamination from the furnace in which the parts were annealed.

### 3.3. Surface Roughness Analysis

The research results for the calculated main roughness parameters are shown in Table 12. The measurements for all specimens were conducted in the same manner. For the specimens obtained via the FDM/FFF process, the surface roughness parameters were significantly higher than in the case of their SLA counterparts. This phenomenon is typical for these two technologies in the production of parts with the use of conventional materials. The sintering process caused an increase in the surface roughness in the SLA parts (about 40%) of the R_a_ parameter, while in the case of the FDM/FFF specimens, this phenomenon was the opposite—the R_a_ parameter decreased from almost 14 μm to 9.71 μm (about 30%).

### 3.4. Hardness Measurement

Hardness measurements were conducted for different loads depending on the specimen type. However, it was not possible to perform Vickers hardness measurements for Specimens 1, 2, and 3, as these specimens exhibited very low hardness levels similar to the polymeric parts. This phenomenon was mainly caused by the presence of the polymer matrix before the final sintering process. The measured hardness values for Specimens 4 and 5 are presented in Table 13.

**Figure 8:**
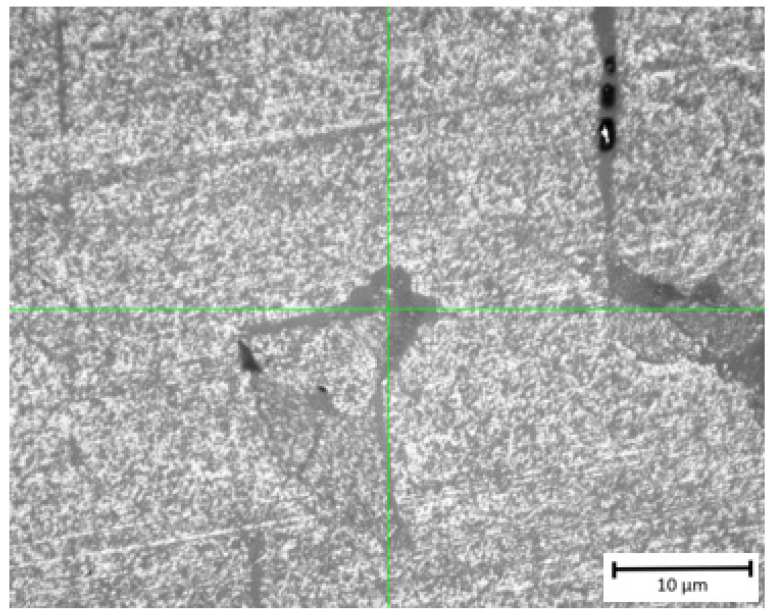
Indentation with visible crack during the analysis of Specimen Series 5.

The obtained result for Specimen Series 4 could be treated as preliminary due to the presence of numerous cracks that occurred at the HV1 load. Such a result indicates a significantly lower hardness level in comparison to conventionally made ceramic parts. The average hardness value for Specimen Series 4 was 3.207 ± 0.783 GPa. Visibly higher hardness values were registered for Specimen Series 5, where the hardness value reached 19.73 ± 0.683 GPa. At the same time, these specimens displayed very high hardness because it was possible to measure the densified material without pores close to the indentations. However, at the interface of the two layers, the measured hardness was lower, and some cracks appeared around the indentations around this area (Figure 8). As a result, the indentation was not well defined, and measuring the diagonal lines of the indentation was challenging, which could affect the precision of this analysis. The measured hardness value aligns with the data provided by the filament manufacturer, who states that the hardness of Al_2_O_3_ ceramics falls within the range of 17–20 GPa. It is worth noting that the obtained result for the alumina specimen produced by FDM/FFF is even higher than the values reported for conventionally manufactured alumina, which has a hardness of approximately 18 GPa. Due to the high brittleness of the specimen, it was not possible to measure the hardness at a higher load to obtain more precise results.

### 3.5. Compression Test Results

**Figure 9:**
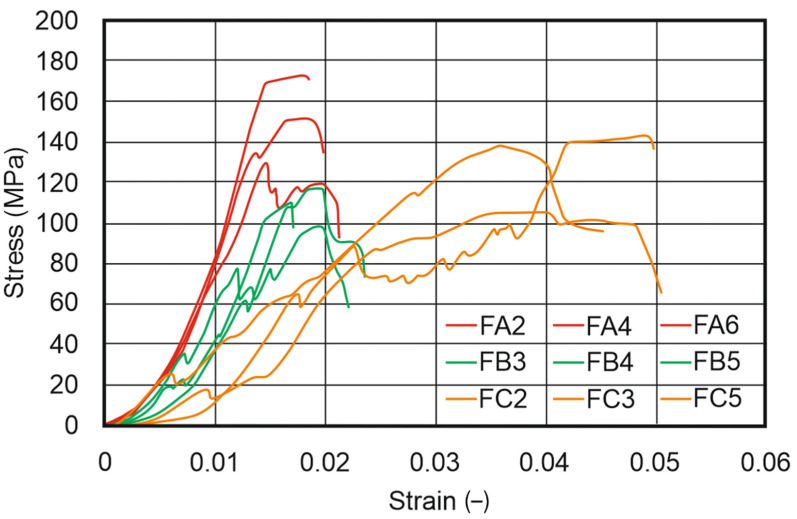
Compression test results for FDM/FFF specimens (Specimen Series E). Curve descriptions are kept from the Figure 1 order.

**Figure 10:**
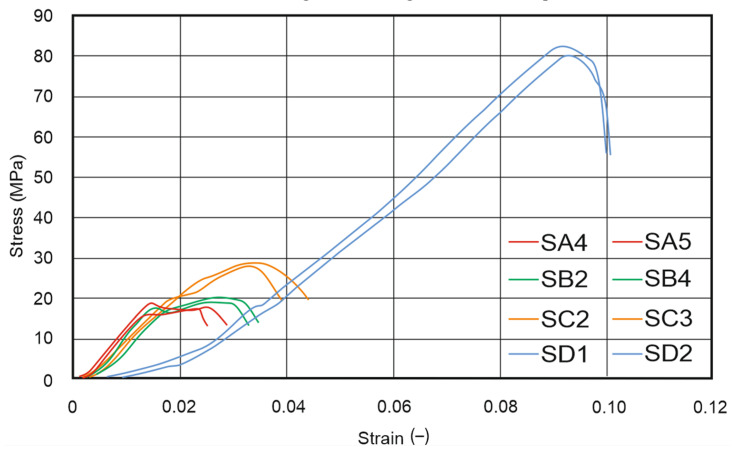
Compression test results for SLA specimens (Specimen Series 4). Curve descriptions are kept from the Figure 1 order.

Based on the conducted experimental tests, stress–strain curves were determined, and parameters such as ultimate compressive strength “R_c_”, strain at compressive strength “ε_Rc_”, and Young’s modulus under compression “E_c_” were calculated. The obtained results are shown in Figure 9 and Figure 10, and the exact, calculated results are included in Table 14. For all tested parts, two extreme results were removed and are not included in the attached charts. This was especially important in the case of the FDM/FFF specimens, where the brittleness of the specimens was significantly higher. Additionally, in the case of the FDM/FFF parts, analyzing the smallest cylindrical specimens (“D” series in Figure 1b) was not possible due to a lack of options for the proper preparation of the top and bottom surfaces. What is more, small defects in the mentioned surfaces could not be removed during specimen preparation and significantly affected the course of the curves shown in Figure 9.

In the case of SLA specimens, the course of all curves was very repeatable, which proves the stability of the obtained material. There are significant differences in the final results between the same size specimens (i.e., FA series and SA series), which was on average 7.5 times higher in FDM/FFF specimens. 

## 4. Summary

Based on the conducted comparison of two different materials available for SLA and FDM/FFF technologies, it was possible to determine the differences between those two technologies, which is useful for finding the potential application of each technology. In comparison to FDM/FFF, SLA-made ceramic parts were characterized by better dimensional accuracy, greater surface roughness, smaller density, about a five-times lower hardness, and worse compression strength. Such results indicate that SLA-made parts would be better for applications that will not apply significant loads into the material structure—e.g., geometrically complex thermal shields or high-aesthetic representative parts exposed to very high temperatures (without significant temperature fluctuations). On the other hand, FDM/FFF parts are much more suitable for industrial applications, where a high dimensional accuracy is not as important and, at the same time, high material hardness and good compression properties are needed. Based on the obtained results, there are several possible paths of further research. One of the most promising is analyzing the influence of using different heat treatments and other postprocessing (e.g., surface treatment or hot isostatic pressing).

## 5. Conclusions

Within the scope of this study, composite parts based on silicon oxide and aluminum oxide ceramics were produced using two printing methods, SLA and FDM/FFF. These elements were subjected to post-processing with various sintering parameters, which significantly reduced the negative effects of artifacts that occurred during AM. All specified objectives were achieved, allowing for the analysis of the conducted research and a comparison of the applied manufacturing methods and types of heat treatment.

The results of the conducted research led to the following conclusions:

1. The composites produced using the FDM/FFF technique exhibited similar microstructures before and after heat treatment. The sintering process did not affect the shape of the grains, only their size. Grain size increased with temperature.
2. For the SLA manufacturing method, sintering influenced the final microstructure. Specimens after printing displayed irregular grain shapes, but as the temperature increased, the grains fused, forming a solid structure that made it impossible to outline individual grains and measure their size.
3. The tested materials exhibited high purity, and sintering did not affect their phase composition. All specimens were single-phase, as confirmed by the results of the diffraction analysis. Minor impurities detected by EDS analysis likely originated from contamination of the furnace.
4. Significant shrinkage was not observed in the extracted FDM/FFF specimens, and none of the specimens exceeded the allowable value specified by the manufacturer. The extraction process was successful, enabling further processing without the occurrence of cracks.
5. Specimens produced using the SLA technique had significantly higher porosity compared to their FDM/FFF counterparts. Microstructural investigations revealed that, under the same sintering parameters, SLA specimens exhibited much greater porosity than FDM/FFF specimens. The average pore volumes demonstrated that the FDM/FFF-printed specimens had larger voids, but their contribution was not significant enough to affect the final material properties. What is more, porosity decreased with increasing temperature. The porosity of the FDM/FFF specimens was largely dependent on the printing process and parameters such as printing speed and temperature. In the case of the SLA technique, reducing the porosity and improving the functional properties of these elements can be achieved through hot isostatic pressing (HIP).
6. With increasing sintering temperature, specimens produced by both methods exhibited significantly higher hardness. However, not all specimens could be subjected to hardness measurements. The FDM/FFF-printed specimens showed the highest hardness, reaching approximately 20 GPa. This value was even greater than the hardness of ceramics produced by conventional methods.
7. The FDM/FFF specimens displayed significantly greater roughness compared to those printed using the SLA method. In the case of FDM/FFF, the average roughness decreased with increasing temperature, whereas for SLA, higher sintering temperatures led to increased roughness. In the FDM/FFF technique, higher roughness is inherent to the material formation during manufacturing. This parameter can be reduced by applying finishing processes such as grinding or polishing.
8. Compressive strength tests revealed significant brittleness of FDM/FFF specimens in comparison to their SLA-made counterparts. This had a significant effect on the surface quality and stability of compression test curves.

## Figures and Tables

**Table 1:** Table of contributions to the present state-of-the-art in the AM of ceramics.

Authors	Used AM Technology	Short Description of the Research
Manière et al. [13]	SLA	A description of anisotropic shrinkage reduction by using post-processing with the use of light-curable resins in the sintering process. Formulation of the relationship between the composition of the ceramic resin and the complexity of the final processing. Ceramic resins used in SLA technology require several components to achieve the desired rheological properties.
Bove et al. [14]	SLA	Description of the selection process of an appropriate solvent or dispersant and its influence on the dimensional accuracy and mechanical properties. Employment of various accompanying process parameters in SLA manufacturing (such as resin mixing or drying the printed object). The initial preparatory phase should involve powder drying at a variable temperature of 120 to 200 °C for 6–10 h to remove any remaining moisture.
C. Hinczewski et al. [15], A. Tsetsekou et al. [16], G.A. Brady et al. [17], T. Chartier et al. [18], K.J. Jang et al. [19]	SLA	Development of correct proportions of each component of the ceramic resin, including photoinitiators, solvents, dispersants, and ceramic powders. In research works [15,18,19], the authors described additional methods for preparing ceramic suspensions for SLA technology.
D. Nötzel et al. [20]	DLP	Using digital light processing (DLP) for production with the use of zirconium oxide and aluminum oxide with a high density of approximately 97–99% and hardness values of 13.1 and 17.5 GPa, respectively, comparable to counterparts obtained using conventional methods.
Y. Gao et al. [21]	DLP	Obtaining ceramic structures with very good properties made of aluminum oxide and bioactive glass during the DLP process. Such a material combination allowed for achieving a relative density above 90% and mechanical strength comparable to conventionally processed specimens.
Esteves et al. [22]	DLP	Fabrication of 3D ceramic biocomponents with interconnected micro- and macrostructures using DLP from a suspension composed of aluminum oxide powders and hydroxyapatite, which was added to the aluminum oxide to enhance its bioactivity.
K.H. Tan et al. [23], C. Gao et al. [24], A.T. Clare et al. [25], C. Shuai et al. [26], J. Liu et al. [27], L. Zhu et al. [28], J. Delgado et al. [29], H.H. Tang [30], J. Ma et al. [31]	SLS	Development of additional binders with lower melting temperatures, which are either coated onto or mixed with the base material. This approach allows the laser beam to heat the surface of the powder bed, causing the binders to melt and creating a glassy binding phase for the ceramic particles. The binder can be organic, such as polymers [23,24,25,26], or inorganic, such as low-melting materials based on glass and metals [27,28,29,30,31].
Z. Chen et al. [32]	SLS	Using additional post-processing, which involves high-temperature sintering, evaporated the binder, resulting in ceramic elements. Structural ceramics were almost fully densified to achieve optimal mechanical properties and improve their porosity. The initial attempts to produce 3D ceramic components using the SLS technology were conducted for mixed powder systems based on Al_2_O_3_. To decrease the melting point of aluminum oxide, a low-temperature binder was added to the powder in the form of secondary particles of ammonium phosphate NH_4_H_2_PO_4_ (190 °C) and boron oxide B_2_O_3_ (460 °C). As a result, 3D ceramic parts with good accuracies were successfully produced.
Shahzad et al. [33]	SLS	An AM of aluminum oxide (Al_2_O_3_) with additional post-processing in the form of hot isostatic pressing (HIP). By employing a suitable laser exposure strategy during the SLS process, densities of up to 62% at the maximum level were achieved. However, the final density after binder removal and sintering in a furnace increased only 51% of the theoretical density. On the other hand, the use of HIP allowed densities to reach 63–64%.
Deckers et al. [34]	SLS	Combination of SLS with HIP and infiltration as techniques to increase the density of Al_2_O_3_ ceramics. After the final processing, the volume increased from 34% to 83%, and the final density increased from 63% to 88%.
Grossin et al. [35]	SLS	Achieving higher densities in parts by optimizing the particle packing in the powder bed. Two primary factors contributing to powder bed spreading are the flowability and packing density of the powder particles. To control these factors, spherical and densely packed powder particles should be employed. The use of particles with non-uniform shapes can lead to non-uniform regions in the spread layers and reduce flowability and packing density.
Bertrand et al. [1]	SLM	AM production of lattice-shaped objects from five types of pure zirconia and yttria powders. It was demonstrated that the use of the smallest possible particle size is required to achieve pure fused ZrO_2_-Y_2_O_3_ layers. The density of the printed elements was relatively low, reaching approximately 56%, and further sintering in a conventional furnace did not increase the object’s density. It was also observed that the laser partially melts the ceramic particles and solidifies the structure.
Shishkovsky et al. [36]	SLM	Examination of the microstructure and phase composition of porous ceramics produced using the SLM method. A mixture of yttria-stabilized zirconia (90 wt% ZrO_2_, 10 wt% Y_2_O_3_) and aluminum in a ratio of 4:1 was prepared for the study. The macro- and microstructures of the surfaces of the tested specimens exhibited relatively high density with visible pores and cracks. Moreover, it was demonstrated that laser melting with high irradiation speeds allows for the attainment of a homogeneous structure with an even distribution of stabilizing phases.
K. Kandananond [37], L. Zheng et al. [38], W. Li et al. [39]	FDM/FFF	Development of filament production with the use of composite fibers and densely loaded ceramic particles (up to 60% volume) into thermoplastic binders. Obtained material is similar to the conventional FDM/FFF filament, and the printed ceramic parts underwent debinding and sintering to achieve proper densification. The initial application of FDM/FFF for ceramic fabrication was described using binder-filled systems of Al_2_O_3_ and Si_3_N_4_.
N. Eliaz et al. [40]	FDM/FFF	Unsatisfactory sintering densities (75–90%) were observed due to the presence of defects such as void spaces in the sintered elements. Current applications of ceramic FDM/FFF mainly involve the production of bioceramic components and photonic bandgap lattice structures.
M.L.M. Sistiaga et al. [41], R. Anitha et al. [42]	FDM/FFF	Analyzes the influence of size and distribution of ceramic particles, fiber dispersions, binders, additives, viscosity, consequently, and the flexibility of continuous fibers on the FDM/FFF process.

**Table 2:** Physical properties of conventionally made Al_2_O_3_ [43].

Chemical Formula and Purity	Al₂O₃ 99.5%	Al₂O₃ 99.9%
Vickers hardness (GPa)	18	18
Brittle-like cracking resistance (MPa·m^0.5^)	4	4
Max. usage temperature (°C)	1300	1500
Thermal expansion coefficient (×10^−^⁶/°C)	8.5 (at 1000 °C)	8.8 (at 1000 °C)
Thermal conductivity (W/(m·K))	30	33
Thermal shock resistance (°C)	200	200

**Table 3:** Physical properties of the Al_2_O_3_ FDM/FFF filament (after sintering) [44].

Material Type	Al_2_O_3_ Filament
Content of organic ingredients (% of total mass)	19.5
Filament density before sintering (g/cm^3^)	2.533
Density after sintering (g/cm^3^)	3.85–3.96
Vickers hardness (GPa)	17–20
3D printing temperature (°C)	from 155 to 170
Printing speed (mm/s)	from 5 to 30
Sintering temperature (°C)	1540 °C
Sintering atmosphere	Air
Linear thermal expansion coefficient (10^−6^K^−1^)	8.5
Thermal conductivity (W/mK)	20–30
Electrical conductivity	insulator
Linear shrinkage (%) (X/Y printing direction)	LSX/Y = 19.0 → SF = 1.235
Linear shrinkage (%) (Z printing direction)	LSZ = 21.5 → SF = 1.274
Extraction time (h)	24
Extraction temperature (°C)	42
Extraction medium	acetone
Maximum weight loss during extraction (%)	9.8

**Table 4:** Properties of SLA-printed ceramic resin parts before sintering [45].

Ultimate Tensile Strength (MPa)	5.1
Young’s modulus (GPa)	1.03
Elongation at break (%)	1.4
Bending stress at break (MPa)	10.27
Heat deflection temperature at 1.13 MPa (°C)	74.4

**Table 5:** Properties of SLA-printed ceramic resin parts after sintering [45].

Young’s Modulus (GPa)	50
Bending stress at break (MPa)	33.5
Cold compression strength (MPa)	72.2
Shear modulus (GPa)	21.9
Poisson’s ratio	0.140
Density (g/cm^3^)	1.9

**Table 6:** SLA specimens’ heat treatment program.

Step No.	Heating Time (min)	Temperature (°C)	Total Time (min)
1	0	0	0
	240	240	240
2	480	240	720
	60	300	780
	60	300	840
3	333	1271	1173
4	5	1271	1178
5	60	900	1238
	450	0	1688

**Table 7:** FDM/FFF specimens’ heat treatment program.

Step No.	Start Temperature (°C)	Target Temperature (°C)	Time (min)
1	20	1000	240
2	1000	1540	270
3	1540	1540	120
4	1540	20	(cooled in the furnace)

**Table 8:** Samples series descriptions used during the research.

Specimen Description	Material Condition
A	Al_2_O_3_ produced via FDM/FFF (green part)
B	SiO_2_ produced via SLA (green part)
C	Al_2_O_3_ produced via FDM/FFF (brown part)
D	SiO_2_ produced via SLA, sintered at 1271 °C (fully ceramic part)
E	Al_2_O_3_ produced via FDM/FFF, sintered at 1540 °C (fully ceramic part)

**Table 9:** True density and average pore volume of AM specimens at all tested conditions.

Specimen Description	True Density (g/cm^3^)	Average Pore Volume (cm^3^/g)
A	2.576	0.5998 ± 0.0006
B	1.721	0.4081 ± 0.0013
C	2.880	0.6416 ± 0.0012
D	2.306	0.5466 ± 0.0014
E	3.920	0.7351 ± 0.0004

**Table 10:** Average grain size determined by stereological analysis.

Specimen Description	Average Grain Size (μm)
A	0.18
B	2.00
C	0.24
D	n/a
E	0.50

**Table 11:** Chemical composition determined by SEM—EDS analysis.

	Amount of Elements (Wt %)					
Specimen Description	Al	O	Ca	Si	Na	K
A	57.2	42.8	n/a	n/a	n/a	n/a
B	10.3	52.9	n/a	30.8	4.4	1.5
C	58.4	41.6	n/a	n/a	n/a	n/a
D	6.6	50.1	n/a	36.9	4.1	2.4
E	53.6	45.2	1.2	n/a	n/a	n/a

**Table 12:** Surface roughness parameters measured in all tested AM specimens.

Parameter	Value	Image
Specimen Series A		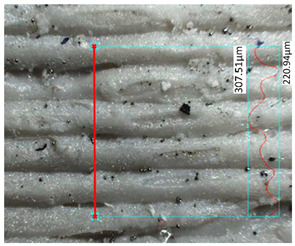
Measured profile length [μm]	1214.43	
Measured profile height [μm]	86.57	
R_z_ [μm]	89.41	
R_a_ [μm]	13.98	
Specimen Series B		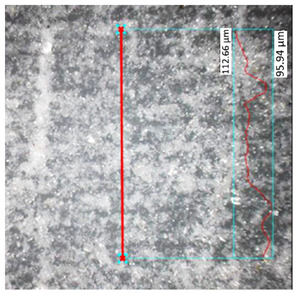
Measured profile length [μm]	1285.22	
Measured profile height [μm]	16.73	
R_z_ [μm]	11.97	
R_a_ [μm]	2.31	
Specimen Series C		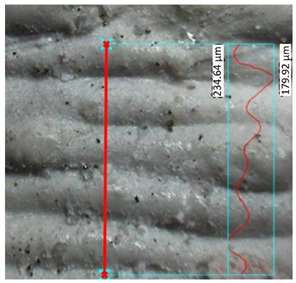
Measured profile length [μm]	1112.69	
Measured profile height [μm]	54.73	
R_z_ [μm]	48.24	
R_a_ [μm]	8.32	
Specimen Series D		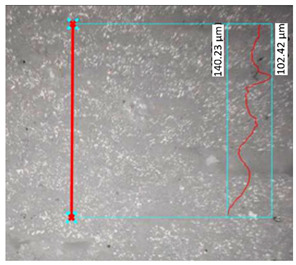
Measured profile length [μm]	957.83	
Measured profile height [μm]	37.80	
R_z_ [μm]	22.15	
R_a_ [μm]	3.91	
Specimen Series E		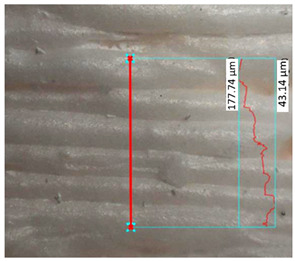
Measured profile length [μm]	1028.61	
Measured profile height [μm]	134.59	
R_z_ [μm]	66.43	
R_a_ [μm]	9.71	

**Table 13:** Vickers hardness measured in all AM specimens subjected to sintering.

Specimen Series Number	Indentation Load	Average Hardness (GPa)
4	HV1	3.207 ± 0.783
5	HV5	19.73 ± 0.683

**Table 14:** Compression test results for all AM specimens.

SLA (Specimen Series 4)				FDM/FFF (Specimen Series 5)			
	Rc [MPa]	Ec [GPa]	eRc [-]		R_c_ [MPa]	E_c_ [GPa]	ε_Rc_ [-]
SA4	19.227	1.883	0.016	FA2	151.841	9.37	0.019
SA5	19.100	1.976	0.023	FA4	168.502	19.379	0.014
Average	19.1635	1.9295	0.0195	FA6	130.053	10.937	0.015
Std. dev.	0.0635	0.0465	0.0035	Average	150.132	13.229	0.016
SB2	20.024	1.652	0.029	Std. dev.	15.743	4.396	0.002
SB4	21.324	1.411	0.029	FB3	116.248	6.009	0.018
Average	20.674	1.5315	0.029	FB4	101.932	13.432	0.015
Std. dev.	0.650	0.1205	0.000	FB5	98.336	12.410	0.019
SC2	28.206	1.358	0.035	Average	105.505	10.617	0.017
SC3	28.345	1.558	0.033	Std. dev.	7.737	3.285	0.002
Average	28.2755	1.458	0.034	FC2	105.703	9.506	0.040
Std. dev.	0.0695	0.1	0.001	FC3	137.102	7.181	0.041
SD1	82.28	1.08	0.93	FC5	138.181	5.603	0.036
SD2	84.71	1.089	0.087	Average	126.995	7.430	0.039
Average	83.495	1.0845	0.5085	Std. dev.	15.062	1.603	0.002
Std. dev.	1.215	0.0045	0.4215				

## Data Availability

Data are contained within the article.
